# Paving the Path to Patient-Centered Healthcare in Africa: Insights From a Student Led Initiative

**DOI:** 10.5334/aogh.4250

**Published:** 2024-04-05

**Authors:** David Jolly Muganzi, Catherine Misango Namara, Timothy Mwanje Kintu, Linda Atulinda, Raymond Bernard Kihumuro, Bonaventure Ahaisibwe, Victor M. Montori

**Affiliations:** 1 Patient Centered Care movement Africa (PaCeM-Afro), Kampala, Uganda; 2 The Patient Revolution, Inc, United States; 3 African Center of Excellence in Bioinformatics and Data Intensive Sciences, Makerere, University, Kampala, Uganda; 4 Seed Global Health, United States; 5 Knowledge and Evaluation Research Unit, Mayo Clinic (Rochester, Minnesota), United States

**Keywords:** Patient-Centered Care, Africa, Student-led initiative

## Abstract

Patient-centered care (PCC) is a key domain of healthcare quality. Its importance is driven by evidence-based medicine, the predominance of chronic conditions requiring self-care, and the recognition of the priority of patient goals, values, priorities, and preferences in determining care plans. This article emphasizes the urgent need for Africa to develop PCC and a workforce committed to its implementation, as well as highlights an initiative by African medical students to champion PCC continent-wide. Embracing this transformative approach presents Africa with an unprecedented opportunity to improve care for each person. Through a comprehensive exploration of unique strategies and considerations in African health professions education, this viewpoint seeks to spark dialogue and inspire action towards a future where patient-centered care is the foundation of healthcare delivery in Africa.

## Introduction: The need for patient-centered care in Africa

Patient-Centered Care (PCC) is defined as being respectful and responsive to individual patient preferences, needs, and values, and ensuring that patient values guide all clinical decisions [1]. PCC encompasses more than just the interaction between patients and healthcare workers; it includes the patient’s entire experience within the healthcare system. For healthcare to be truly patient-centered, it must integrate various dimensions of care. These include the interpersonal dimension (relationships), clinical dimension (provision of care) and the structural dimension (system features) [2]. The core principles of PCC are critical to this integration. These principles include effective communication between clinicians, patients and their families, shared clinical decision support, coordination and continuity of care, infrastructure that adequately accommodates patient, clinician and family needs, and appropriate access to care with minimum waiting time, among others [2, 3].

PCC as a component of healthcare quality offers numerous benefits for patients, families, and payers, making it an important consideration in advancing global health [4]. Adopting a PCC approach not only improves patient satisfaction and perception of quality of care, but also contributes to improved adherence to treatment and overall health outcomes. A randomized controlled trial focused on patients with type 2 diabetes demonstrated that additional PCC training for healthcare professionals resulted in better doctor-patient communication, higher satisfaction, and lower hemoglobin A1c levels [5]. PCC also plays a vital role in reducing malpractice complaints, improving clinician satisfaction, and reducing health disparities [6]. However, implementing PCC in Africa presents unique challenges. Overcoming deeply rooted cultural barriers that favor a biomedical focused, disease-centric, technology-driven, and doctor-dominated approach to healthcare remain significant hurdles.

### Challenges and gaps in implementing patient-centered care in Africa

The realization of PCC in Africa is contingent upon the coordinated synergistic efforts of three key stakeholders: patients, health workers, and policymakers—particularly African governments [7]. However, the realization of PCC on the continent has been hindered by various obstacles, often stemming from how these stakeholders behave.

We start by examining the role of the patient. Patient participation is important in implementing PCC practices, particularly when it comes to shared decision making. Effective PCC necessitates active involvement from both patients and clinicians. Without this collaboration, services may fail to address the specific needs of individuals, hindering the development of effective care plans and limiting treatment outcomes. However, there are significant barriers to achieving this level of patient participation. Predominantly, these are hierarchical cultural norms in Africa that elevate physician knowledge above all and undermine patient involvement, suggesting that the physician’s expertise alone is adequate for treatment decisions. This issue is compounded by limited patient education and self-efficacy, as well as minimal opportunities for patients to actively engage in decision-making or influence the shape of healthcare services.

On the side of health workers, understanding patient preferences is vital for providing PCC. A study in rural Tanzania surveyed women’s delivery care preferences, and identified kindness by doctors, medical knowledge, and modern equipment as crucial factors [8]. Such findings emphasize the need for available, competent, and compassionate healthcare professionals for effective patient engagement. However, sub-Saharan Africa (SSA) faces a significant challenge: a low patient-to-health worker ration. This shortage restricts the time clinicians can dedicate to meaningful interactions with patients, understanding their preferences, and involving them in decision-making processes. The same health systems are further strained by staff shortages, low staff motivation and inadequate supervision [9]. Furthermore, there is inadequate staff training in communication, limited accountability in the provision of care, and poor infrastructure to ensure patient confidentiality and protection of privacy [10, 11].

It is important to recognize that the deficiencies in the health system, which hinder the implementation of PCC in Africa, are both a result of, and further exacerbated by, gaps in health professionals’ education. A prevalent issue in medical training across the continent is the persistence of a ‘doctor knows best’ mentality. This paternalistic approach, often demonstrated by teachers and role models to medical students, typically sidelines patient preferences, needs, and expectations [12]. During this critical period of learning for young health professional students, cornerstones of PCC, such as excellence in communication, cultural competence, and attention to psychosocial aspects, have been reported to erode during medical training [13]. As a result, these health professionals (in training) struggle with patient-centered communication, finding it challenging to engage with patients effectively in this manner [14].

Furthermore, while the global North has recognized the importance of training in achieving PCC, health professional training in Africa remains rooted in outdated and colonial-era models [15]. While this lag has not been fully investigated, it may be a symptom of policy inertia and lack of advocacy for reform toward PCC. This lack of training may be contributing to the limited implementation and impact of grassroots efforts such as patient charters. Integrating PCC principles early in healthcare professionals’ training ensures their adoption as part of their professional identity and may create the conditions for the adoption of PCC as a practice without a myriad of separate initiatives. By prioritizing early integration of PCC principles and practices in healthcare professional curricula, Africa can foster a healthcare workforce that embraces patient-centered values and practices for the foreseeable future. These challenges have created a pressing need to reform health professions education. Such reforms should aim at preparing future health professionals to implement PCC effectively across diverse healthcare contexts.

### PaCeM-Afro’s contribution to bridging the gap in health professional education

The World Health Organization designated 2021 the Year of the Health and Care Worker—focusing on improving healthcare outcomes by enhancing the availability, accessibility, and capacity of health and care workers to deliver exceptional people-centered integrated care. A remarkable idea emerged to contribute to this transformative journey. A collective of impassioned African health workers in training united under this common purpose, giving birth to the Patient-Centered Care Movement Africa (PaCeM-Afro). The genesis of this movement gained accelerated momentum with the endorsement and recognition from the frontline health workers’ coalition on the sidelines of the 74th World Health Assembly.

Guided by an unwavering vision to advance PCC, this movement aspires to drive the realization of PCC in Africa. This is only most sustainably possible by cultivating generations of health professionals who are not only deeply committed to PCC but are also equipped with the knowledge and skills to construct and lead patient-centered health systems in Africa. Through its various activities and initiatives, PaCeM-Afro builds on the best practices from several innovative training initiatives implemented in Africa before, including alignment to local priorities, country ownership, and institutional capacity building [16]. PaCeM-Afro’s programs seek to expand students’ knowledge of PCC, foster thought leadership within their communities, and advocate for increased visibility of PCC among students, healthcare professionals, communities, and policymakers. The hope is that this collective effort will ignite a sustainable, cross-generational transformation in healthcare quality throughout Africa.

PaCeM-Afro is a vibrant and dynamic multidisciplinary community of practice, encompassing health professional students from diverse backgrounds across Africa, united by their dedication to championing PCC. This community leverages a multidisciplinary, multinational student-led Secretariat to guide its activities and contribute to its mission and vision. The Secretariat ensures the movement’s strategic direction aligns with best practices in PCC, and is supported by an advisory board comprised of experienced healthcare professionals committed to PCC from across Africa and beyond.

In recognition of the fact that there is no one-fits-all approach in advocating for PCC across the different African communities, the secretariat has supported the creation of semi-autonomous chapters across universities offering health professional courses. These chapters have attracted health professional students who are inspired to change the status quo of health care on the continent. The chapters are led by a dedicated chapter ambassador, who collaborates closely with the secretariat to drive impactful initiatives, in line with the overall objectives of the movement.

**Figure 1:**
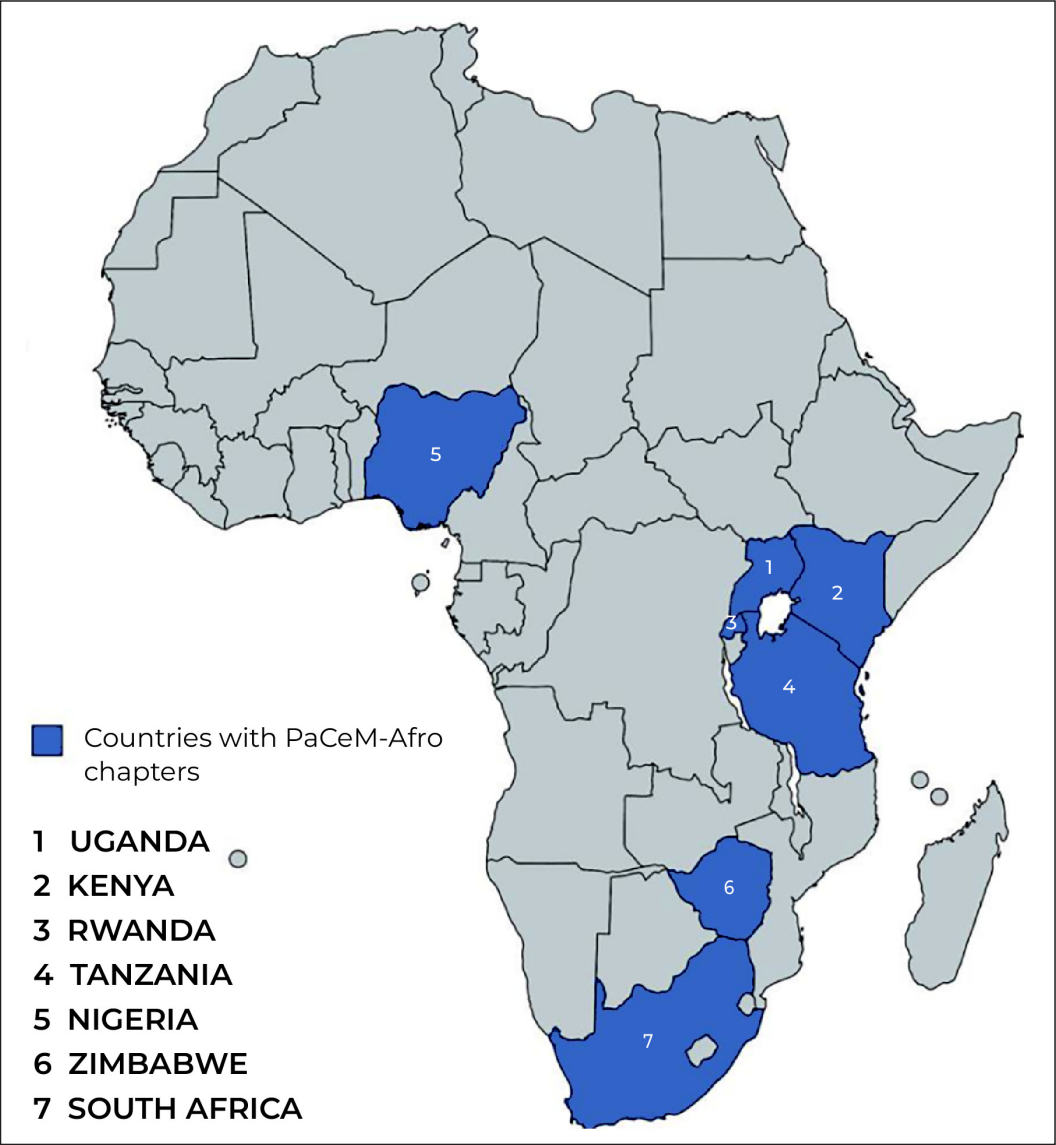
Countries with PaCeM-Afro chapters.

Over the past two years, the movement has welcomed over 1,000 health professional students from more than 10 countries, and has successfully established 14 chapters in seven African countries (Figure 1). These chapters have since engaged in activities championing and advocating for PCC, including workshops, symposia, webinars, patient education sessions at health facilities, radio talk shows, digital campaigns, and quizzes about PCC. These sessions are facilitated and led by healthcare professionals and community leaders who champion PCC. The PCC campaign held in March 2022 had 494 volunteers from across Africa. Through roundtable discussions, articles, infographics and videos, the campaign had a reach of over 30,000 people. The different chapter activities have had at least 800 students actively participate, promoting knowledge, skills, and commitment to PCC. The exponential growth in the membership and activities of PaCeM-Afro is testament to the unwavering commitment of African health professional students to improving health and care outcomes.

PaCeM-Afro has also established a research team comprised of 15 health professional students from 7 African countries. The team is assessing the status of PCC in health professional curriculum, country-level policy alignment with PCC, and the state of knowledge, attitudes, and practices about PCC in current students. This work should help document gaps and barriers to PCC and draw lessons on how to overcome them.

In 2023, PaCeM-Afro organized the inaugural Patient-Centered Care Conference in Africa, in which 108 attendees from 8 countries and 11 disciplines heard form 36 speakers through lectures and panel discussions. Through strategic partnerships with renowned institutions—including the International Federation of Medical Students’ Associations: Africa and the World Continuing Education Alliance—PaCeM-Afro has broadened its reach and amplified its impact. PaCeM-Afro members have presented and advocated for PCC at local and international conferences, such as the Consortium of Universities for Global Health 2023 Conference and the Towards Unity for Health Africa 2022 Regional Conference.

## Conclusion

The imperative to prioritize PCC within all facets of the health system is undeniable, as it is pivotal for crafting effective, responsive, efficient, and sustainable healthcare systems. The role of health professional students in this transformation cannot be overstated. They represent a tremendous opportunity to cultivate a health workforce that is both competent and compassionate in delivering patient-centered care. At the forefront of this transformative journey is PaCeM-Afro. This community is dedicated to reshaping the healthcare landscape in Africa towards a more patient-centered approach. Through a range of impactful activities, innovative research studies, awareness campaigns, and collaborative efforts, PaCeM-Afro is not just advocating for change but actively inviting other stakeholders to join in building a healthcare ecosystem that is fundamentally patient-centered. This collective effort is crucial for championing the establishment of patient-centered health systems throughout the continent, marking a new era in African healthcare.
